# Bioinformatics Analysis of Estrogen-Responsive Genes

**DOI:** 10.1007/978-1-4939-3127-9_4

**Published:** 2015-07-04

**Authors:** Adam E. Handel

**Affiliations:** 1 Department of Physiology, Anatomy and Genetics, University of Oxford, South Parks Road, Oxford, OX1 3QX UK; 2 Weatherall Institute of Molecular Medicine, University of Oxford, Headley Way, Oxford, OX3 9DS UK

**Keywords:** Estrogen, ChIP-seq, Transcriptomics, Gene targetGene targets, Bioinformatics

## Abstract

Estrogen is a steroid hormone that plays critical roles in a myriad of intracellular pathways. The expression of many genes is regulated through the steroid hormone receptors *ESR1* and *ESR2*. These bind to DNA and modulate the expression of target genes. Identification of estrogen target genes is greatly facilitated by the use of transcriptomic methods, such as RNA-seq and expression microarrays, and chromatin immunoprecipitation with massively parallel sequencing (ChIP-seq). Combining transcriptomic and ChIP-seq data enables a distinction to be drawn between direct and indirect estrogen target genes. This chapter discusses some methods of identifying estrogen target genes that do not require any expertise in programming languages or complex bioinformatics.

## Introduction

Gender disparities are associated with the risk of multiple diseases [1]. Estrogen is clearly associated with the risk of many gynecological malignancies but also has a role in modulating aspects of autoimmunity [2–4]. Therefore, understanding estrogen-regulated gene pathways is critical to understanding the pathophysiology of many diseases. This in turn requires an understanding of the dynamics of estrogen-regulated gene expression and the binding of *ESR1* and *ESR2*, the nuclear receptors through which estrogen exerts much of its effect [5].

Identifying estrogen-responsive genes is an apparently simple problem. The obvious method to use is to profile gene expression in the presence or absence of estrogen [6]. This can be performed either by expression microarray, which involves the use of tiling oligonucleotide probes and identifying the targets of RNA hybridization, and RNA-seq, which involves fragmenting RNA in cells and sequencing cDNA reverse transcribed from these RNA fragments [7]. However, depending on the time course used in transcriptomic experiments, this will identify both direct estradiol target genes and secondary genes modulated by those direct target genes (*see***Note****1**).

Chromatin immunoprecipitation with massively parallel sequencing (ChIP-seq) is a technique that allows for the genomic localization of nuclear receptor binding [7, 8]. This technique uses the formation of formaldehyde cross bridges between DNA and proteins bound to nucleic acid, followed by selective sequencing of DNA fragments that have been immunoprecipitated by an antibody directed against a protein of interest. In case the of estrogen, fragments that are immunoprecipitated with antibodies against ESR1 or ESR2 can be compared with fragments immunoprecipitated by nonspecific antibodies (input control) or fragments can be compared between samples pre- and posttreatment with estrogen. Stimulation with estrogen (or estrogen receptor agonists) can be problematic as, just as in the case of transcriptomics, the duration of stimulation can be an important consideration in capturing different aspects of receptor binding (*see***Note****2**). Remodeling of the chromatin architecture and the 3D structure of the genome are likely to be complex and time-dependent processes, which mean that the snapshot of estrogen receptor occupancy afforded by ChIP-seq may not always be representative of the underlying biology (*see***Note****3**) [9, 10].

This chapter concentrates on basic methods of identifying direct estrogen target genes by combining transcriptomic and ChIP-seq data. The methods by which nuclear receptors are assigned to gene targets in particular cell types either in vitro or in vivo are continuously evolving both due to the availability of new techniques and the increasingly encyclopedic datasets available on genomic architecture in a multitude of cell types (*see***Note****4**). However, here we provide a series of simple workflows that rely heavily on the Galaxy web interface and the Genomic HyperBrowser that are effective ways of identifying a set of estrogen direct gene targets with relatively high confidence [11–15]. These offer the distinct advantages that no prior knowledge of bioinformatics or programming languages is required for their use.

The first approach described explains how to identify genomic intervals for a series of genes differentially expressed in response to estrogen treatment and intersect these with ESR1 ChIP-seq-binding→ sites. The second uses a purpose-built bioinformatics tool called BETA that is able to use transcriptomic and ChIP-seq data to identify potential ESR1 target genes [16]. Both assume that the user has transcriptomic and ChIP-seq data available from their cells of interest treated with estrogen and that these data have been processed to obtain differentially expressed genes (DEGs) and significant ChIP-seq peaks. Previous chapters in this series explain how to accomplish this [17, 18].

## Materials

1. Modern laptop or personal computer running a modern operating system with at least 60 GB of hard disc memory and 4 GB of RAM.
2. Basic software for manipulating spreadsheets (e.g., Microsoft Excel or OpenOffice Calc).
3. Internet browser software (e.g., Internet Explorer, Mozilla Firefox or Google Chrome).
4. A high-speed Internet connection.

## Methods

### The Genomic HyperBrowser

1. Register for the Genomic HyperBrowser (https://hyperbrowser.uio.no/hb/).
2. Prepare ChIP-seq and transciptomic datasets for upload. ChIP-seq files should be a set of tab-delimited genomic coordinates corresponding to each peak in the format:
  Chromosome Start Stop
Transcriptomic files should be a list of differentially expressed genes (DEGs) as ENSEMBL IDs (*see***Note****1** for methods for converting between different forms of gene ID). The datasets used for this demonstration are the ESR1-binding site data (actually ChIP-chip data) from Hurtado and colleagues and transcriptomic data from Hah and colleagues (thresholded at *q* < 0.05) [6, 19].
3. Firstly upload the ChIP-seq data as shown in Fig. 1.

**Fig. 1:**
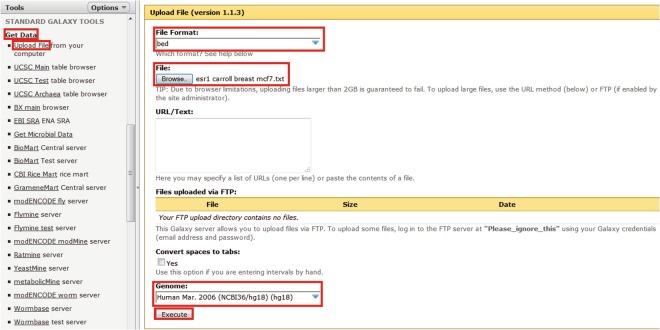
Uploading files to the Galaxy/Genomic HyperBrowser server. Select “Get Data > Upload files,” select the correct file type (in this case “bed” for ChIP-seq data or “txt” for transcriptomic data), select the file location using the “browse” button, select the correct genome build, and then select “Execute”
4. Next use “Generate Tracks > Generate segment track from gene IDs” to obtain genomic intervals from the ENSEMBL gene IDs of DEGs. These should be uploaded into the tool as a series of comma-separated values as shown by the demo data. If necessary genomic intervals can be lifted from one genome build to another by the “Lift-Over > Convert genomic coordinates” tool.
5. Use “Operate on Genomic Intervals > Get flanks” to extend gene regions by a pre-specified number of bases in each direction (Fig. 2). A suitable distance might be 5 kb, which is analogous to the upstream region extension in the gene ontology tool GREAT [20].

**Fig. 2:**
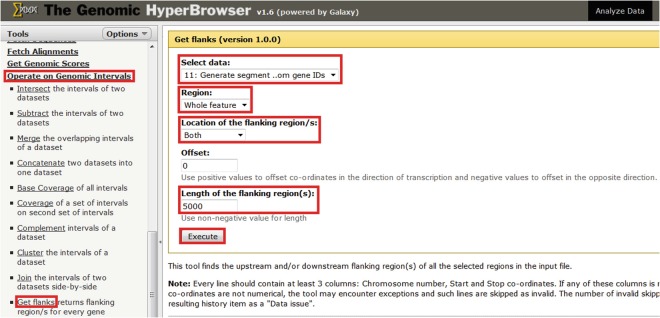
Generating a track of regions flanking differentially expressed genes. Select “Operate on Genomic Intervals > Get flanks,” select the desired track, select the subset of the region to flank (in this case “whole region”), select whether to extend flank from the upstream, downstream or both sides of regions, decide on the length of flanking regions, and then select “Execute”
6. The original intervals and flanking regions should then be concatenated into a single track using “Operate on Genomic Intervals > Concatenate” and then merged with “Operate on Genomic Intervals > Merge.”
7. An important sanity check is to ensure that estrogen receptor binding is enriched near estrogen DEGs. The Genomic HyperBrowser allows one to calculate the enrichment of estrogen receptor-binding sites with the intervals generated above (i.e., within 5 kb of estrogen DEGs). Figure 3 illustrates this process. It is possible to use the Genomic HyperBrowser to calculate an empirical *p*-value for this overlap using the same tab as for enrichment analysis but selecting “Category: Hypothesis testing,” “Overlap?,” a suitable null model (e.g., “Preserve segments (T2), segment lengths and inter-segment gaps (T1); randomize positions (T1) (MC)”) and the number of permutations (e.g., for publication quality *p*-values ~10,000 permutations would be recommended). The region and scale tab is also important as this determines in which areas of the genome randomized tracks can fall. Leaving it at its default value (all chromosome arms) is adequate for the current sanity check. There is significant overlap between ESR1-binding sites and estrogen DEGs (2.14-fold, *p* < 10^−4^), which suggests that there are likely to be plausible direct estrogen targets amongst the transcriptomic dataset. Note that analyses are only conducted on bed files, and so if the track of interest is not offered by the Genomic HyperBrowser as a potential track for analysis then edit that track to ensure that the track type is “bed.”

**Fig. 3:**
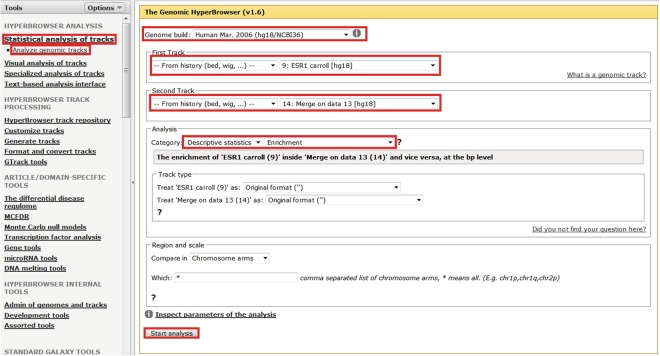
Performing enrichment analysis. Select “Statistical analysis of tracks > Analyze genomic tracks,” select the genome build, select that each track for analysis will be from your history and then select the appropriate track, select “Descriptive statisticDescriptive statistics,” select “Enrichment,” and then select “Start analysis”
8. Identifying potential direct estrogen targets is simply a matter of joining estrogen DEGs (±5 kb) to ESR1-binding sites (Fig. 4).

**Fig. 4:**
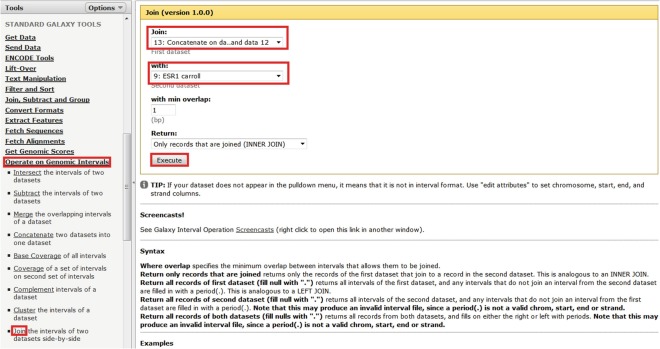
Joining two tracks side by side. Select “Operate on Genomic Intervals > Join,” select the required tracks, and then select “Execute”
9. The resultant output can be pasted into a spreadsheet program and filtered to obtain unique gene IDs and their respective ESR1-binding sites.

### BETA

1. Register for Galaxy/Cistrome (http://cistrome.org/ap/). This tool integrates transcription factor-binding sites with the degree of differential gene expression to predict high-confidence direct targets.
2. Prepare ChIP-seq and transcriptomic data for upload. Again, the ChIP-seq data should be a tab-delimited file in the format:
  Chromosome Start Stop
  RNA-seq data can either be directly uploaded as Cuffdiff or LIMMA output [21, 22] or formatted as a tab-delimited file with columns corresponding to gene ID, direction of change (e.g., *T-*score) and significance (e.g., FDR). Ensure that all data are present as text (gene ID) or numerical data. Some spreadsheet programs can format data in ways that will cause BETA to crash (e.g., substituting dates for numerical values).
3. Start the BETA tool running after selecting the appropriate parameters (Fig. 5). The BETA tool is available through “Integrative Analysis > BETA-plus: Binding and Expression Target prediction and motif analysis.” For an initial analysis, it is recommended to leave as many settings at their default values as possible. Subsequently these can be altered to test how robust the results are to changes in, for example, the distance threshold of ESR1-binding sites to DEGs.

**Fig. 5:**
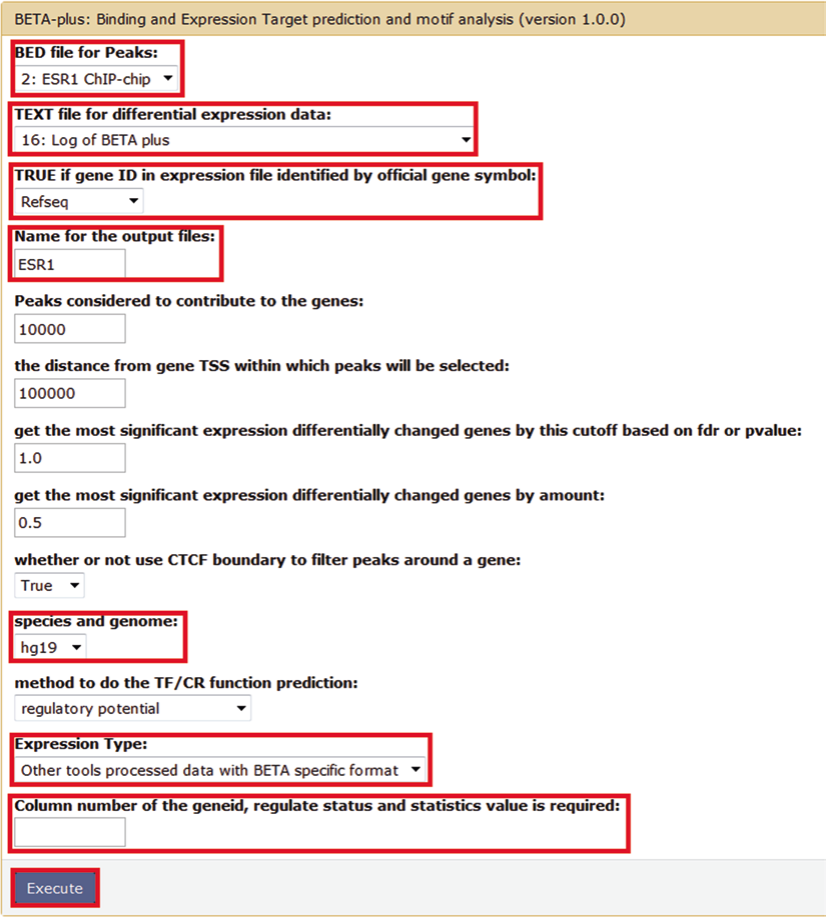
Performing BETA-plus analysis. Select the track containing the ChIP-seq data, select the track containing the transcriptomic data, select the type of gene ID used (RefSeq or gene symbol), input the prefix for output files, select the genome build, select the type of transcriptomic data (i.e., the format of the track selected earlier), if the transcriptomic data is in a custom format insert a comma-separated list of numbers referencing which column is the gene ID, the direction of expression change and the significance measure (e.g., if this was a track with three columns, the first of which was the gene ID, the second of which was the log_2_ fold change and the third of which was the FDR, this would be 1,2,3). Finally select “Execute”
4. The output files then produce direct target predictions. These are described below:
  - BETA functional prediction on ESR1 ChIP-chip: A graph showing the relationship between functional rank and the number of direct targets and an associated *p*-value for up- or downregulated genes.
  - BETA direct targets prediction on up regulated genes: A table of up-regulated gene targets detailing the rank product score (derived from the significance score provided in the transcriptomic dataset).
  - BETA direct targets prediction on down regulated genes: A table of downregulated gene targets detailing the rank product score (derived from the significance score provided in the transcriptomic dataset).
  - Uptarget associated peaks: A list of peaks with the associated up-regulated gene target, the distance to the target gene, and a functional score.
  - Downtarget associated peaks: A list of peaks with the associated downregulated gene target, the distance to the target gene, and a functional score.
  - Motif analysis on target regions: An html output file detailing top motifs detected for multiple comparisons along with associated statistical scores.
  - A series of detailed motif analysis outputs: The statistical data for the above file.
  - Log of BETA plus: This details the input parameters and any errors encountered during the course of the analysis.

## Notes

1. *Converting between different gene IDs*: There are multiple ways of converting between different forms of gene ID (e.g., gene symbol, RefSeq, ENSEMBL). One simple way is to use the Table Browser function in UCSC Genome Browser (http://genome.ucsc.edu/) (Fig. 6) [23]. It is possible to convert gene IDs between multiple types of gene ID using sequential conversions.

**Fig. 6:**
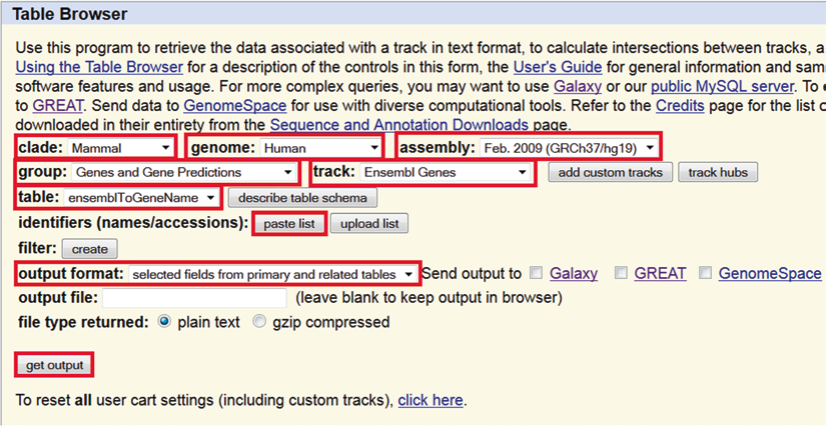
Convert gene IDs from one form to another. Select the required clade, the species and the genome build. Select the “Genes and Gene Predictions” group of tables, the appropriate track (e.g., Ensembl genes), the desired table (this will be one that maps the gene ID one has to the gene ID one requires), paste the list of gene IDs (this will be checked for unknown IDs by the system), select that output should be “selected fields from primary and related tables,” and then select “get output.” On the resulting screen it is possible to select the desired fields to obtain the gene ID of interest
2. *Considerations for experimental design*: Replicates are essential for ChIP-seq and transcriptomic analysis when attempting to distinguish biologically meaningful variation from noise. As mentioned in the *introduction*, if using stimulation with estrogen or an estrogen receptor agonist, it is vital to decide on the time scale of stimulation to ensure that the correct cross section of binding and transcriptomic changes are sampled.
3. *Limitations of current bioinformatic methods*: There are several important limitations to consider when interpreting lists of direct gene targets. The output is only as good as the data input into the model in the first place. This can be an issue particularly for ChIP-seq datasets, which are noisy and frequently irreproducible in the main between different studies nominally using the same material and methods [24]. However, new methods for calling ChIP-seq peaks, such as irreproducibility discovery analysis, which attempt to leverage power from replicates, may help to alleviate this problem [25]. Another limitation is that distance thresholds applied in calling direct gene targets are linear, whereas it is clear that the 3D structure of chromatin is important in determining which binding sites interact with which genes [9]. Methods for considering 3D structure in enrichment analyses are available through the Genomic HyperBrowser but the interpretation of such data is not straightforward [26].
4. *Further functional annotation of direct gene targets*: As mentioned above, many of the thresholds used are rather arbitrary and so it can be informative to include other forms of functional annotation to hone down a list of potential gene targets to ones of higher confidence. ESR1 binding sites are more likely to be consistent between different ChIP datasets if they possess a classical ESR1 recognition motif or are located in a region of open chromatin (as assessed by DNase-seq) [24]. BETA will supply a measure of motif enrichment within the peaks supplied and estrogen receptor motifs should be significantly enriched in direct gene targets. Motif scanning software such as FIMO can be used to assess whether specific ESR1-binding sites contain estrogen receptor recognition motifs and this can assist in the selection of high-confidence gene targets [27]. There is a wealth of data on chromatin state or RNA polymerase II binding in many cell types with and without estrogen stimulation available from databases like UCSC Genome Browser. These can be downloaded and intersected with candidate direct gene targets just as in Subheading 3.1 to select high-confidence direct gene targets. Gene targetGene targets containing an estrogen receptor recognition motif, a DNase hypersensitivity peak, and with a nearby RNA polymerase II ChIP-seq peak in addition to an ESR1 ChIP-seq peak are highly likely to be direct estrogen targets [28].
